# Effect of Exposure to a Mixture of Organic Solvents on Hearing Thresholds in Petrochemical Industry Workers

**Published:** 2014-10

**Authors:** Ziba Loukzadeh, Ahmad Shojaoddiny-Ardekani, Amir Houshang Mehrparvar, Zohreh Yazdi, Abolfazl Mollasadeghi

**Affiliations:** 1 *Industrial Diseases Research Center, Shahid Sadoughi University of Medical Sciences, Yazd, Iran.*; 2 *Department of Occupational Medicine, Qazvin University of Medical Sciences, Qazvin, Iran.*

**Keywords:** Hearing loss, Noise, Noise-induced hearing loss, Ototoxicity, Organic solvent, Pure-tone-audiometry.

## Abstract

**Introduction:**

Hearing loss is one of the most common occupational diseases. In most workplaces, workers are exposed to noise and solvents simultaneously, so the potential risk of hearing loss due to solvents may be attributed to noise. In this study we aimed to assess the effect of exposure to mixed aromatic solvents on hearing in the absence of exposure to hazardous noise.

**Materials and Methods:**

In a cross-sectional study, 99 workers from the petrochemical industry with exposure to a mixture of organic solvents whose noise exposure was lower than 85 dBA were compared with 100 un-exposed controls. After measuring sound pressure level and mean concentration of each solvent in the workplace, pure-tone-audiometry was performed and the two groups were compared in terms of high-frequency and low-frequency hearing loss. T-tests and Chi-square tests were used to compare the two groups.

**Results:**

The mean hearing threshold at all frequencies among petrochemical workers was normal (below 25 dB). We did not observe any significant association between solvent exposure and high-frequency or low-frequency hearing loss.

**Conclusion:**

This study showed that temporary exposure (less than 4 years) to a mixture of organic solvents, without exposure to noise, does not affect workers’ hearing threshold in audiometry tests.

## Introduction

Noise-induced hearing loss is one of the most common occupational diseases globally in occupational environments (1). Although exposure to noise above 85 dB at work is the most important cause of hearing loss (1,2), it has been proposed in various studies that different chemicals such as metal fumes, asphyxiant gases, and organic solvents (e.g. toluene, xylene, styrene, n-hexane, ethylene tetrachloride, and carbon disulfide) may be ototoxic substances (3).

A study in 2002 showed that 30 million workers in Europe were exposed to unauthorized noise and 10 million further workers were exposed to harmful chemicals including industrial solvents. Many of these workers had simultaneous exposure to noise and solvents (4). Although accurate statistics are not available concerning the number of workers exposed to noise in Iran, it can be assumed that the prevalence of noise exposure in Iran is considerable (5). Solvents are chemicals with extensive usage in many industries, but there is little information about their effects on human health (1). In 1980, Rebert et al. showed evidence of the ototoxicity of organic solvents in rat models (6). Barregard and Axelsson reported that in four workers simultaneously exposed to noise and solvents, the intensity of hearing loss was greater than the level expected for noise alone (7).

Some studies in animals and humans have shown that exposure to organic solvents is associated with an increased risk of noise-induced hearing loss. Solvents cause the outer hair cells to become more vulnerable and more susceptible to injury due to noise(8,9); on the other hand they reduce the protective effect of the middle ear through suppression of protective reflexes such as acoustic reflex, and lead to the increased penetration of noise (10). Therefore, in the simultaneous exposure to noise and solvents, the same level of noise causes greater injury to hearing. There are few studies reporting the effect of solvents on hearing in humans, and the results that have been published are controversial. This may be due to the difference in type and concentration of the solvent, exposure to mixed solvents, method of solvent use, and route and duration of exposure to solvents (2,3).

Epidemiologic studies have shown a 2-5 fold risk of hearing loss after exposure to organic solvents (9,11,12). Some studies in rat models showed that organic solvents such as styrene and toluene damage the cochlear and retrocochlear components (13,14).

Assessment of the effect of organic solvents in humans is more difficult than in animals, because higher doses are used in animal studies in order to assess the relationship. Furthermore, workers are often exposed to a mixture of solvents with different chemical structures and different concentrations and, most of the time, exposure to organic solvents and noise are simultaneous; hence differentiation of their effect is difficult (13).

In most workplaces where workers are exposed to solvents, they are concurrently exposed to noise as well. Therefore, the potential risk of hearing loss is mostly attributed to noise, which is the more common factor and has a higher probability of being associated with hearing loss. In this study we assessed the effect of exposure to mixed aromatic solvents on hearing in the absence of exposure to hazardous noise.

## Materials and Methods

This cross-sectional study was conducted in workers at an Iranian petroleum distillates-producing company in 2009. All employees who worked in areas with significant exposure to mixed organic solvents (equivalent concentration or Em>1) for more than 6 months [2] and without workplace exposure to hazardous noise (≥85 dBA) were enrolled. Workers who were exposed to non-occupational noise as well as those with a positive medical history of diabetes mellitus (15), hypertension, head trauma, head surgery, acoustic trauma, ototoxic drug consumption (loop diuretics, aminoglyco- sides, antineoplastics and salicylates), chronic otitis media, conductive hearing loss in pure-tone audiometry, or exposure to organic solvents or loud noise in a previous or second job or in a non-occupational form were excluded from the study. 

Among 99 petrochemical workers, 14 workers had permissible exposure to organic solvents (Em<1), while two were excluded from the study because of chronic otitis media and one because of diabetes mellitus. Eighty-two exposed subjects with high exposure (Em>1) were enrolled the study. The average work day was 8 hours per day. Most workers did not use respiratory protection devices and a few workers used organic vapor respirator very irregularly.

With regard to exclusion criteria, 100 male subjects who were referred for pre-placement evaluations from May to October 2009 to the occupational clinic were selected by simple random sampling as control group. Each worker completed a checklist to determine medical history for exclusion criteria. 

A questionnaire including age, duration of employment in current job, smoking and alcohol consumption was completed for each subject. An informed consent form was signed by each subject. This study was approved by medical ethics committee of Shahid Sadoughi University of Medical Sciences.

Sound pressure and organic solvents had been measured during annual industrial hygiene assessments of the factory, which was performed 1 month before the study by an industrial hygiene institute. Workplace noise level was measured using a sound-level meter TES-1358 (Taiwan) recording in dBA, in 52 stations of workers positioned at heights of 91 cm and 151 cm in the sitting and standing manner, respectively, according to the National Institute for Occupational Safety and Health (NIOSH) recommended method (16). Then, equivalent sound levels (Leq) were calculated according to ISO 9612 (17).

Workplace organic solvents included benzene, toluene, and xylene and were measured using the NIOSH method (18). Breathing zone samples were collected on activated charcoal tubes at eight positions. Tubes were connected to SKC model-226-01 pumps with a constant flow rate of 100 ml/min, and the duration of the sampling time was 8 working hours. Exhaled air was desorbed by carbon disulfide and analyzed by gas chromatography with a flame ionization detector according to NIOSH method number 1501. The following formula (19) was used to assess the exposure level to the mixture of solvents: Em= C1/L1 + C2/L2 + … + Cn/Ln

In this formula, Em, C and L represent equivalent concentrations of the mixture of organic solvents, mean concentration of organic solvents in the workplace, and permissible exposure level, respectively. The permissible exposure level was considered according to ACGIH-TLV, 2008 (20,0.5, and 100 ppm for toluene, benzene and xylene, respectively) (20). After measuring the mean concentration of each solvent in the workplace environment and calculation using the aforementioned formula, Em>1 was considered significant exposure in the workplace.

Pure-tone audiometry (audiometer: Interacoustic, AC40, Denmark) was performed in an acoustic chamber by an expert audiologist for all subjects. Audiometry was performed by an expert audiologist (blinded to the study) in a sound-isolated chamber, which met the requirements of the American National Standards Institution (ANSI) 2004 (21). Biologic calibration was performed daily. Air conduction was assessed at 500, 1000, 2000, 3000, 4000, 6000, and 8000 Hz frequencies (2) and bone conduction at 250–4000 Hz frequencies. High- frequency hearing threshold (average hearing threshold at 3, 4 and 6 KHz) as well as low-frequency hearing threshold (average hearing threshold at 0.5, 1 and 2 KHz) were evaluated (2). The presence of high or low-frequency hearing loss was defined as average hearing thresholds greater than 25 dB at both high and low frequencies (2,22,23).

SPSS software version 17 (SPSS, Inc., Chicago, IL) was used for statistical analysis. To compare continuous variables between groups by estimating the mean ±SD, we used an independent t-test. A Chi-square test was used to compare hearing loss prevalence in both exposure and control groups. Logistic regression analysis was used to eliminate potential confounders (age, work duration, and smoking) and to test the association between exposure to mixed organic solvents and hearing loss. P≤0.05 was considered statistically significant.

## Results

Demographic details of the petrochemical workers and control group are shown in Table 1. Controls were significantly younger than the cases. Six cases (7.36%) and four controls (4%) were smokers, but there was no significant difference between these groups (P= 0.47). None of the subjects consumed alcohol.

**Table 1:** Demographic data of study groups

	**Exposed (n =82)** **mean ± SD** *	**Control (n =100)** **mean ± SD** *	**P-value**
Age (year)	28.21 ± 3.78	25.98 ± 4.87	0.003
Work duration(year)	3.67 ± 2.61	0.05 ± 0.4	<0.0001

* SD, Standard Deviation

Noise exposure at all sites was lower than 75 dBA. According to Table 2, the concentration of aromatic solvents at all study sites was Em>1. Table 2 shows those workplaces that have a high exposure to organic solvents and the number of workers at each site.

**Table 2:** Mean levels of aromatic solvents measured and number of workers at the study sites

Study site	Number of workers (%)	Concentration (ppm) Benzene Toluene Xylene TLV=0.5 ppm TLV=100 ppm TLV=20 ppm			Em	Leq (dBA)
Quality control	10 (12.2%)	2.71	79.8	21.7	9.63	75
Oil tanker loading	29 (35.4%)	2.71	79.8	21.7	9.63	75
Facilities unit	5 (6.1%)	2.71	79.8	21.7	9.63	*7*4
Fuel position worker	38 (46.3%)	1.52	4.97	2.79	3.32	75

Em**, **The equivalent exposure for the mixture of solvent exposure

Table 3 shows the comparison of hearing loss between study groups according to the type of hearing loss. High-frequency hearing loss was more common in petrochemical workers but this difference was not statistically significant (P=0.15 and 0.08 for right and left ear, respectively). The prevalence of low-frequency hearing loss in exposed and control groups was not statistically different (P>0.05). 

**Table 3:** Comparison between study groups in the terms of high-frequency and low-frequency hearing loss

**Hearing loss**		**Exposed (n = 82)** **N (%)**	**Control (n = 100)** **N (%)**	**Odds ratio (95% CI)**	**P-value**
High-frequency	RE	4 (4.9%)	0 (0%)	0.95 (0.91-1.12)	0.15
	LE	6 (7.3%)	0 (0%)	0.93 (0.87-1.01)	0.08
Low-frequency	RE	0 (0%)	0 (0%)	-	-
	LE	1 (1.2%)	0 (0%)	0.99 (0.96-1.02)	0.99

To control the effects of confounder variables (i.e., age, work duration, and smoking) on the association of solvent exposure and hearing loss, logistic regression was used (Table.4). After elimination of confounding factors, no significant correlation between solvent exposure and hearing loss was observed. There was a significant association between age and hearing loss (P= 0.03).

**Table 4:** Logistic regression analysis for adjusting of confounders in left ear high-frequency hearing loss

**Confounders**	**Adjusted OR**	**95%CI**	**P-value**
Exposure to solvents/yes	0.00	-	0.99
Age/ years	1.18	1.02-1.37	0.03
Work duration/ years	0.99	0.74-1.31	0.93
Smoking/ yes	0.00	-	0.99

**Fig 1:**
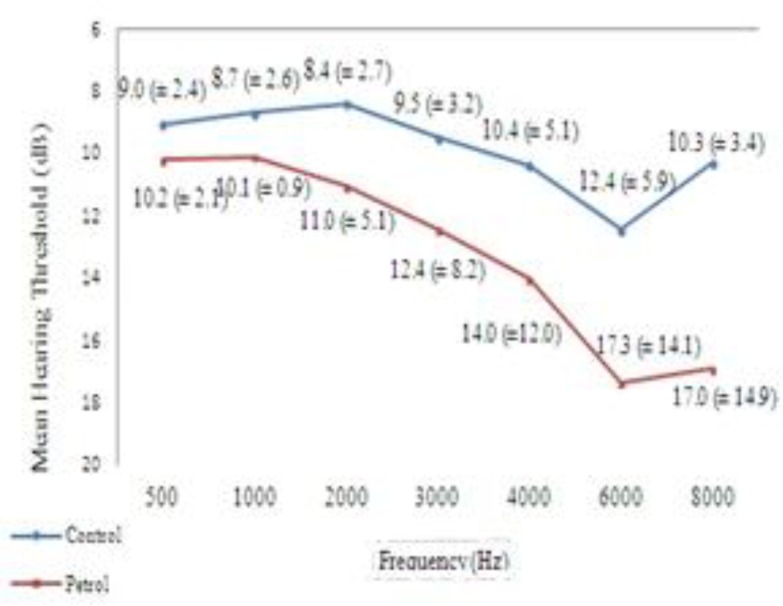
Average of hearing thresholds in the right ear among petrochemical workers and the control group

**Fig 2:**
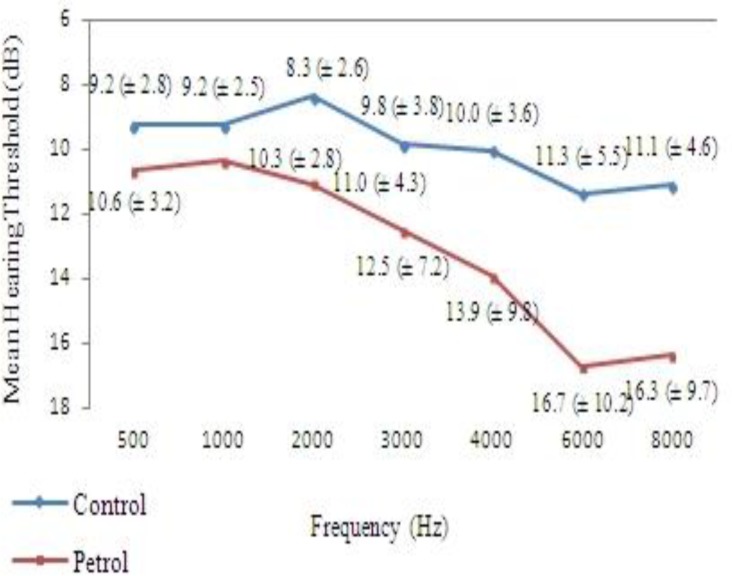
Average of hearing thresholds in the left ear among petrochemical workers and the control group

The mean hearing threshold in both ears was normal (<25 dB) at high and low frequencies (Fig 1,2), but mean hearing thresholds at 6 and 8 KHz frequencies were significantly higher than the others in both ears in the exposed group (Table. 5).

**Table 5:** One-by-one comparison of 6 and 8 KHz with other frequencies in the right and left ears in the exposed group.

**Frequency (Hz)**		6000 **Difference (Mean±SD)** **P-Value**		8000 **Difference (Mean±SD)** **P-Value**	
2000	RE	6.28 ± 11.88	<0.0001	5.85 ± 11.94	<0.0001
	LE	5.67 ± 9.65	<0.0001	5.30 ± 10.34	<0.0001
3000	RE	4.94 ± 10.05	<0.0001	4.51 ± 10.76	<0.0001
	LE	4.21 ± 8.97	<0.0001	3.84 ± 9.82	0.001
4000	RE	3.36 ± 7.33	<0.0001	2.93 ± 8.12	0.002
	LE	2.80 ± 7.12	0.001	2.43 ± 8.86	0.015
6000	RE	-	-	0.43 ± 5.73	0.502
		-	-	0.37 ± 5.26	0.530

RE: Right Ear, LE: Left Ear

## Discussion

The results of this study show that among workers with a mean work duration of 3.7 years, exposure to a mixture of aromatic solvents at levels exceeding permissible levels, without exposure to hazardous noise, might not affect the hearing threshold in pure-tone-audiometry. Other studies investigating the association between the short-term (≤4 years) exposure to a low-concentration mixture of chemicals (<10 ppm for each constituent of the mixture) did not increase the risk of hearing loss (13,24), consistent with the current study; however intermediate concentrations of these chemicals increased the risk of hearing loss (11). In workers exposed to high levels of the mixture of organic solvents (much greater than the permissible levels), a linear dose-response relationship has been reported between the exposure level, risk of hearing loss, and hearing threshold at high frequencies, especially 8000 Hz (25). 

A study of oil refinery workers showed that in those who were exposed to benzene, toluene, xylene, or ethylene in a level lower than TLV, the risk of hearing loss was increased 2.4 times compared with those without exposure to the solvents (26). In another study in the printing industry, frequency of hearing loss among workers exposed to organic solvents was higher than that among other workers (18% vs. 8%). The results of most studies on humans and animals show that exposure to solvents causes permanent hearing loss. Most studies have shown that solvent-induced hearing loss, with or without exposure to noise, predominantly affects high frequencies (consistent with our research) or may affect a wide spectrum of frequencies from 1000 to 8000 Hz (2,11,27). In a study of petrochemical workers, simultaneous exposure to noise and solvents affected both the 6 KHz and 8 KHz frequencies (3). 

The effect of concurrent exposure to noise and organic solvents is controversial. In a study by Jacobson et al, the effect on hearing of simultaneous exposure to noise and organic solvents was no greater than the effect of exposure to noise alone (28). Although some studies have assessed the pure effect of organic solvents on hearing, most workers are simultaneously exposed to a mixture of solvents and noise, which may have an additive or synergistic effect on hearing. Indeed, most studies have shown a synergistic effect (11,29-31). Organic solvents change the structure of the outer hair cells and cause them to become more sensitive to the effect of noise; therefore, simultaneous exposure to organic solvents and acoustic energy has a more potent effect on the cochlea (32). Exposure to noise and solvent individually may be below permissible levels, but their combination can affect hearing (29). In studies investigating workers with concurrent exposure, noise intensity was the most important factor causing hearing loss (13,27,30,33,34). 

The mechanism of hearing loss due to organic solvents is not fully understood. Most studies on humans show cochlear and retrocochlear damage due to exposure to organic solvents (27,30,33,34). Gopal evaluated seven subjects with exposure to industrial solvents. All subjects showed retrocochlear damage, although two showed a normal hearing threshold at all frequencies (1).

Ameno showed that the brain/blood concentration ratio of toluene is highest in the brain stem region of those with exposure to toluene compared with individuals without exposure. These individuals had a significant disturbance in speech understanding, but their audiograms were normal (35). Therefore, it may be that audiometry is not sufficiently sensitive for detecting the hearing effects of solvent. Thus, in our study, despite a normal hearing threshold at all frequencies in audiometry, evaluation of retrocochlear structures by auditory brainstem response or contra-lateral acoustic reflex, as well as speech and high-frequency tests showed some hearing disturbances due to exposure to organic solvents. 

There are a number of similarities and differences among solvents, with the results of animal studies showing that styrene has the most damaging effect on hearing (14). In our study, workers had no exposure to styrene. In contrast, some studies reported no association between exposure to styrene and hearing loss (36). It has been documented that susceptibility to organic solvents varies according to the individual (29).

Our study has some limitations. First, it was a cross-sectional study, and may therefore underestimate the effect of occupational exposures due to the healthy worker effect, and does not allow demonstration of a causal relationship. We were not able to perform biologic monitoring. The results of this study are based on one-point measurements of noise and organic solvents, with no calculation of working lifetime exposures. However, it is worth noting that the workplace environment, including ventilation, work tasks and working conditions, have not changed dramatically over the past few years according to available documents in company, while measured values (including noise and organic solvents) across previous measurements from recent years have not changed greatly (although the measurements were performed one month before the study by an industrial hygiene institute).

There is a need for more studies to show which individuals are more susceptible to the hearing effects of solvents, as well as studies to find the critical level of exposure to solvents which may cause hearing impairment. More studies are needed to show whether a hearing conservation program is required for workers only exposed to organic solvents.

## Conclusion

The findings of this study show that exposure to a mixture of aromatic solvents among workers with short duration in the current job (less than 4 years) might not affect the hearing threshold in audiometry. However, other studies, especially longitudinal ones, are needed to prove this issue. 
